# Eye-Tracking as a Screening Tool in the Early Diagnosis of Autism Spectrum Disorder: A Systematic Review and Meta-Analysis

**DOI:** 10.3390/jcm14248801

**Published:** 2025-12-12

**Authors:** Cristina Tecar, Lacramioara Eliza Chiperi, Bianca-Elena Iftimie, Livia Livint-Popa, Emanuel Stefanescu, Sur Maria Lucia, Nicu Catalin Draghici, Dafin Fior Muresanu

**Affiliations:** 1 RoNeuro Institute for Neurological Research and Diagnostic, 400364 Cluj-Napoca, Romania; cristina.pantelemon@umfcluj.ro (C.T.); livia.popa@umfcluj.ro (L.L.-P.); emanuel.stefanescu@brainscience.ro (E.S.); nicu.draghici@umfcluj.ro (N.C.D.); dafin.muresanu@umfcluj.ro (D.F.M.); 2 Monza Ares Hospital, 400347 Cluj-Napoca, Romania; 3 Department of Neurosciences, Psychiatry and Pediatric Psychiatry, Iuliu Hațieganu University of Medicine and Pharmacy, 400347 Cluj-Napoca, Romania; biancaiftimie4@gmail.com; 4 Department of Neurosciences, Neurology and Pediatric Neurology, Iuliu Hațieganu University of Medicine and Pharmacy, 400083 Cluj-Napoca, Romania; 5 1st Department of Pediatrics, Iuliu Hațieganu University of Medicine and Pharmacy, 400083 Cluj-Napoca, Romania; sur.maria@umfcluj.ro

**Keywords:** autism spectrum disorder, eye-tracking, diagnostic methods, screening method, neuroplasticity, systematic review, metanalysis

## Abstract

**Background:** Early detection of autism spectrum disorder (ASD) is essential, as the first two years of life represent a critical window of neuroplasticity during which timely interventions can improve developmental outcomes. Traditional diagnostic methods, such as ADOS and ADI-R, rely on caregiver reports and structured observations, limiting ecological validity and accessibility. Eye-tracking (ET) offers a non-invasive, scalable approach to assess early atypical gaze patterns. **Objectives:** This systematic review and meta-analysis synthesized evidence on the diagnostic accuracy of ET for early ASD detection and its potential as an adjunctive screening tool. **Methods:** A comprehensive search of PubMed, Scopus, Web of Science, Medline, and the Cochrane Library identified studies published between January 2015 and July 2025. Eligible studies evaluated ET in infants and toddlers (≤36 months) for early ASD identification, following PRISMA guidelines. **Results:** Out of 513 records, 57 studies were included. Most studies reported reduced fixation on social stimuli, atypical gaze following, and preference for geometric over social images in infants later diagnosed with ASD. Pooled effect sizes indicated a moderate-to-large difference between ASD and typically developing groups in social fixation time (Hedges’ g ≈ 0.65, 95% CI: 0.48–0.82, I^2^ = 58%). Studies integrating machine learning algorithms (*n* = 14) achieved improved sensitivity (up to 89%) and specificity (up to 86%) compared with conventional gaze metrics. **Conclusions:** Overall, ET shows strong potential as an early adjunctive screening method for ASD. Nonetheless, methodological heterogeneity and lack of standardized protocols currently limit clinical translation, underscoring the need for multi-center validation and task standardization.

## 1. Introduction

Autism Spectrum Disorder (ASD) is a neurodevelopmental condition characterized by social communication difficulties, restricted interests, and repetitive behaviors [1]. Early signs of ASD can emerge as early as 4–6 months of age [2,3], yet the median age of formal diagnosis remains approximately 4.5 years [4]. Delayed diagnosis represents a missed opportunity, as the first two years of life constitute a critical period of neuroplasticity during which early intervention can improve cognitive, language, and social outcomes [5].

While clinically relevant, current diagnostic instruments, such as the Autism Diagnostic Observation Schedule (ADOS) and the Autism Diagnostic Interview-Revised (ADI-R) rely heavily on caregiver reporting and structured behavioral observation—conditions that do not fully capture the complexity of real-world social interactions and further delay diagnosis [3]. Moreover, they require extensive training and are time-intensive, which diminishes access in disadvantaged communities.

One of the earliest behavioral markers of children with ASD, observed as early as 2–6 months, are differences in gaze behavior [2]. Eye tracking (ET) is a procedure that can assess gaze behaviors in real time in response to various types of stimuli—social or non-social, static or dynamic [6]. The flexibility of these stimuli provides a closer approximation to real-world social encounters. Gaze behavior is tracked via infrared-based eye-tracking systems and the procedures typically last less than 10 min [3]. Thus, the method is feasible for infants or severely impaired children who cannot participate in more demanding procedures [3,4].

One commonly observed finding of ET studies is that individuals with ASD, compared to typically developing (TD) controls, present reduced fixation on face and eye regions [3,7,8], often associated with a less systematic analysis of facial features [8]. Several studies identified that children with ASD prefer non-social stimuli, focusing more on objects or patterns, such as repetitive or geometric ones [9,10]. Furthermore, children with ASD have difficulties in redirecting attention based on the gaze or gesture of another [6], a necessary function for joint attention [11].

However, other studies have failed to replicate these findings [3,10]. Variables such as age, cognitive ability, and comorbidities can modulate these outcomes [3,6]. Moreover, differences in experimental stimuli and methodology, along with small sample sizes continue to limit cross-study comparability [10,11].

Despite the growing body of research, it remains unclear which gaze-based measures show the greatest robustness across contexts and to what extent between study variability reflects true developmental differences versus methodological heterogeneity. Quantifying the magnitude and consistency of reported effects is therefore necessary before ET can be used reliably as a diagnostic method. Also, standardized stimuli and methodologies must be established to improve cross-study consistency [3,10]. Until then, ET fits the criteria for becoming an adjoining diagnostic and screening step [3,4].

Several attempts were made in order to increase the specificity and sensitivity of ET. One method entails the analysis of both social and non-social stimuli dwell times, resulting in a composite score—ARI, or Autism Risk Index—which has been strongly correlated with ADOS-2 results [3]. Recent studies also integrate artificial intelligence (AI) and machine learning (ML) to extract predictive patterns from gaze behavior [12,13,14], some noting the increase in specificity and sensitivity of diagnostic scores [13,15].

At the point of care, the technical infrastructure required for ET could be largely automated [3,12,16]. Although initial costs for acquiring the hardware and software components could be substantial [3], the implementation at the point of delivery can be managed by trained technicians [4]. This approach entails smaller costs for training the personnel, broader availability, making ET a sustainable option for community-based screening and diagnosis [3,16].

Additionally, its repeatability makes it suitable for longitudinal monitoring of developmental trajectories [7,10,16]. The integration of software and machine learning algorithms allows for objective measures of gaze behavior [12,13,15]. However, replicability remains constrained by methodological heterogeneity [3,10,17].

Given these considerations, a systematic review and meta-analysis is warranted in order to synthesize existing findings, formally assess the magnitude and heterogeneity of gaze-related differences, and examine the influence of the methodological factors across studies.

Accordingly, the present study was guided by the following research questions:

(1) What eye-tracking measures of early social attention differentiate infants and toddlers with ASD or an elevated likelihood thereof from typically developing peers?
(2) What is the magnitude and heterogeneity of gaze-based group differences reported across studies?
(3) To what extent do task paradigms and methodological characteristics contribute to variability in reported findings?
(4) What is the current translational potential of eye-tracking measures, including AI- and ML-based approaches, for early ASD risk stratification?

This systematic review and meta-analysis aims to 1. summarize current evidence on ET-based biomarkers for early ASD detection; 2. quantify the effect size of gaze-based differences between ASD and typically developing infants; and 3. identify methodological and technological challenges for clinical translation of ET.

## 2. Materials and Methods

### 2.1. Search Strategy

A systematic literature search was conducted in five electronic databases: PubMed, Scopus, Web of Science, Medline, and the Cochrane Library. The search covered the period from 1 January 2015 to 28 July 2025. The following combination of keywords and MeSH terms was used for PubMed:

(“Eye Movement Measurements” OR “eye tracking” OR “gaze behavior”) AND (“autism spectrum disorder” OR “autism” OR “ASD”) AND (“Early Diagnosis” OR “early detection” OR “early identification” OR “infants” OR “toddlers”).

Database-specific syntax was adapted for each platform, and a full search strategy is provided in Supplementary Table S1. Reference lists of included studies and relevant reviews were screened manually to identify additional records. Search filters were restricted to English-language original research involving human infants or toddlers aged ≤ 36 months.

This review followed the PRISMA 2020 guidelines. See Supplementary Materials for details. No review protocol was registered in PROSPERO prior to conducting this study, due to the exploratory nature of the project. Future updates of this review will be registered to ensure full transparency.

### 2.2. Eligibility Criteria

Studies were eligible if they met the following criteria: (1) Population: Infants or toddlers aged ≤ 36 months with a confirmed ASD diagnosis, elevated familial likelihood of ASD, or high-risk status based on standardized screening tools. (2) Intervention/Index test: Eye-tracking assessment of gaze behavior, social attention, or related metrics; (3) Comparator: Typically developing or non-ASD control groups, or within-group analyses over time; (4) Outcomes: Quantitative measures relevant to ASD diagnosis, such as fixation time, gaze-following metrics, composite indices (e.g., Autism Risk Index), or diagnostic performance (sensitivity, specificity, area under the curve); (5) Study design: Original research (cross-sectional, longitudinal, or case–control) published in English. Several studies initially appeared to meet the inclusion criteria based on title and abstract but were excluded after full-text review for specific reasons (see Supplementary Table S4).

Exclusion criteria included: reviews, editorials, conference abstracts, and case reports; animal studies; studies lacking relevant quantitative data and non-English publications. These exclusion criteria ensured that only studies directly assessing quantitative eye-tracking metrics for social versus non-social visual attention in infants and toddlers (≤36 months) with confirmed or high-likelihood ASD were retained for synthesis.

### 2.3. Study Selection Process

**Figure 1:**
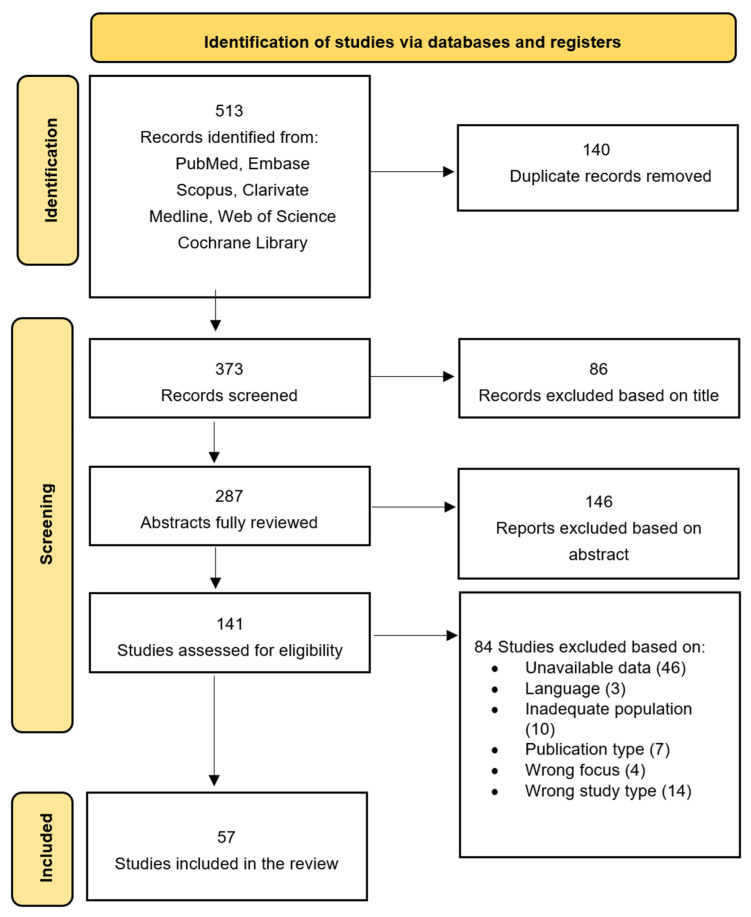
PRISMA flow diagram for new systematic review: selection process of included studies.

To ensure transparency and reproducibility, we predefined explicit decision rules for both title/abstract and full-text screening. Two reviewers independently screened all records. At the full-text stage, each article was independently evaluated according to a structured checklist derived from the eligibility criteria (population, paradigm, outcomes, study design, and data extractability) in a non-blinded manner. Disagreements were resolved through discussion; unresolved cases were adjudicated by a third reviewer. All full-text exclusion decisions were documented with a specific exclusion reason, and exported in detail into Supplementary Table S4. The selection process is summarized in Figure 1 (PRISMA 2020 flow diagram). Coding of study characteristics, classification into diagnostic/predictive/descriptive categories, and extraction of eye-tracking metrics were performed independently by two reviewers, following predefined coding rules. Any discrepancies in extracted data (e.g., outcomes, sample characteristics, effect-size inputs) were resolved through consensus.

After full-text review, 84 studies were excluded for the following specific reasons: (1) Unavailable quantitative data (*n* = 27): studies reporting only qualitative descriptions, lacking extractable fixation metrics, group means, or diagnostic accuracy parameters; (2) Inadequate population (*n* = 18): samples older than 36 months, non-human studies, or studies without ASD/high-likelihood infants; (3) Wrong intervention/index test or wrong focus (*n* = 16): studies using paradigms unrelated to social eye-tracking (e.g., oculomotor randomness, EEG–ET combined tasks, visual search tasks); (4) Wrong outcomes or wrong study type (*n* = 11): studies that did not provide primary eye-tracking diagnostic metrics or used designs incompatible with extraction (e.g., simulation, case reports, methodological notes); (5) Publication type (*n* = 16): reviews, conference abstracts, short communications, editorials, or early online supplements without full data; (6) Language restrictions (*n* = 4): studies published in languages other than English for which no translation was available. All exclusion decisions are documented in Supplementary Table S4, with the exact reason for exclusion listed for each study.

### 2.4. Classification of Study Types

To reduce conceptual ambiguity, all included studies were classified into three predefined categories based on their primary objective:

1. Diagnostic accuracy studies: Studies that directly compared ASD vs. typically developing (TD) or non-ASD comparison groups and reported metrics related to group discrimination, diagnostic accuracy, or effect-size differences in social fixation.
2. Predictive or longitudinal studies: Studies that assessed whether early eye-tracking metrics predicted later ASD outcomes, developmental status, or familial high-likelihood trajectories. These studies typically involved infants with elevated likelihood of ASD (e.g., infant siblings) and reported associations with later clinical endpoints.
3. Descriptive or exploratory studies: Studies that characterized gaze patterns across groups or paradigms without assessing diagnostic performance or predictive value. This includes feasibility studies, paradigm-development studies, and early-phase research using prototype or low-N paradigms.

This classification was used in both qualitative synthesis and interpretation of heterogeneity. Only diagnostic accuracy studies were included in the quantitative meta-analysis. Predictive and descriptive studies were synthesized narratively because their aims, outcomes, and study designs were conceptually distinct from diagnostic accuracy evaluation.

### 2.5. Data Extraction

A standardized extraction form was used to collect data on: study characteristics (authors, publication year, country, design); participant demographics (sample size, age, sex, risk status); eye-tracking equipment and tasks; outcomes (fixation duration, gaze shifts, joint attention metrics, composite indices); diagnostic performance (sensitivity, specificity, AUC); statistical methods and effect sizes.

### 2.6. Quality Assessment

Quality assessment of diagnostic accuracy was performed using the QUADAS-2 tool, evaluating risk of bias and applicability concerns across four domains: Patient Selection, Index Test, Reference Standard, and Flow and Timing. For each included study, domains were rated as ‘low risk,’ ‘some concerns/unclear,’ or ‘high risk.’ Detailed domain-by-domain ratings for all studies are provided in Supplementary Table S3.

### 2.7. Statistical Analysis

#### 2.7.1. Prespecified Analytic Plan

The analytic plan was developed a priori, prior to data extraction, and was based on prespecified hypotheses aligned with the objectives of the review. Specifically, we hypothesized that:

(1) children with ASD would show reduced fixation to social stimuli compared to typically developing controls;
(2) paradigm type (e.g., Geo/Social, dynamic social scenes, joint-attention tasks) would contribute systematically to heterogeneity;
(3) studies using validated, conventional eye-tracking metrics would yield more consistent effect sizes than studies using prototype or ML-enhanced measures.

Based on these hypotheses, we predefined the following analytic rules before reviewing the extracted data: only diagnostic accuracy studies would contribute to meta-analysis; effect sizes would be computed as Hedges’ g for comparability; when studies reported multiple dependent outcomes, they would be aggregated within-study to prevent artificial inflation of precision; predefined subgroup analyses (paradigm category, age group, type of metric) would be performed regardless of statistical significance; predictive and descriptive studies would be excluded from quantitative synthesis, consistent with the conceptual focus on diagnostic performance. These prespecified elements guided all stages of data synthesis, and no analytic decisions were modified based on the pattern of results.

#### 2.7.2. Diagnostic-Accuracy Context

Because most included studies did not report conventional diagnostic metrics (sensitivity, specificity, AUC), effect sizes (Hedges’ g) were used as standardized measures of between-group separation. To align with diagnostic accuracy conventions, we contextualized these effect sizes relative to their expected impact on discrimination performance. Specifically, effect sizes above ~0.80 were interpreted as compatible with large group separations, while smaller effects (<0.50) imply limited diagnostic utility unless paired with high sensitivity/specificity thresholds. This approach enables comparison across heterogeneous paradigms while acknowledging that standardized effect sizes are not substitutes for diagnostic performance metrics.

#### 2.7.3. Meta-Analysis Process

A random-effects meta-analysis was performed to calculate standardized mean differences for social fixation time between ASD and TD groups. Effect sizes were calculated as standardized mean differences (Hedges’ g) to ensure comparability across studies using different social-attention metrics (e.g., percentage of fixation time to faces, eyes, social scenes; dwell time; social/non-social preference ratios). When studies reported multiple eligible social-fixation outcomes, we applied the following strategy:

1. Selection of a single primary outcome per paradigm: For studies using the same paradigm (e.g., Geo/Social, dynamic social scenes), the outcome representing overall social fixation (e.g., % time on faces or eye region) was prioritized.
2. Averaging within-study effects: If a study reported several non-independent social-fixation outcomes within the same task (e.g., eyes, mouth, whole face), these were aggregated into a single composite effect size, following recommended procedures for dependent outcomes.
3. Multiple task paradigms within the same study: When a study included multiple paradigms, we extracted only the outcome that best aligned with the primary aim of this review (i.e., early social attention or social vs. non-social preference), to avoid double-counting participants.

All effect sizes and variances were calculated using reported means, standard deviations. When studies did not report means and standard deviations required for quantitative synthesis, we derived missing values following established procedures. For studies reporting medians and interquartile ranges, means and SDs were estimated. When only standard errors, confidence intervals, or test statistics (t, F) were available, we converted these to SDs using standard formulas. If outcomes were presented only in graphical form, numerical values were extracted using digital plot-reading software (WebPlotDigitizer version 4.6), and SDs were computed from extracted data points. Imputation was performed only when sufficient information existed to support accurate derivation of summary statistics; no assumptions were made when underlying distributional parameters could not be reliably reconstructed. All imputation rules and conversion formulas were applied consistently across studies.

#### 2.7.4. Subgroup Analyses

A random-effects model (restricted maximum likelihood, REML) was used to account for expected methodological heterogeneity across studies. Heterogeneity was quantified using I^2^, with values < 25%, 25–50%, and >50% interpreted as low, moderate, and high heterogeneity, respectively.

To explore sources of heterogeneity, the following prespecified subgroup analyses were performed: (1) ET paradigm: Geo/Social preference tasks; Dynamic social scenes; Joint attention or gaze-following tasks; (2) Age group: ≤18 months; 19–36 months; (3) Machine learning-enhanced metrics vs. traditional ET measures. Due to insufficient reporting, meta-regression could not be performed.

#### 2.7.5. Sensitivity Analyses

We conducted multiple sensitivity analyses: (1) Exclusion of studies rated as high risk of bias based on QUADAS-2; (2) Exclusion of effect-size outliers (>2 SD from the pooled mean); (3) Leave-one-out analyses to assess the stability of pooled estimates.

Publication bias was assessed using funnel plot asymmetry and Egger’s regression test. All analyses were performed using R (version 4.3.2) with the metafor package.

### 2.8. Data Synthesis

To enhance conceptual clarity, results were organized into three complementary subsections. First, ‘main behavioral findings’ synthesize outcomes from traditional eye-tracking and pupillometry paradigms examining group-level differences. Second, studies employing machine learning approaches were reported separately due to their distinct analytic pipelines, classification-oriented objectives, and non-comparability with conventional behavioral outcomes. Finally, meta-analytic results were presented in a dedicated section to describe the quantitative aggregation of effect sizes. This structure reflects the methodological heterogeneity of the literature and allows each domain to be interpreted within its appropriate analytic framework.

## 3. Results

### 3.1. Included Studies

The database search identified 513 records. After removing 140 duplicates, 373 records were screened by title and abstract, and 287 full-text articles were assessed for eligibility. A total of 57 studies met the inclusion criteria and were included in the systematic review and meta-analysis (Figure 1, PRISMA 2020 flow diagram).

### 3.2. Study Characteristics

A summary of individual study characteristics is provided in Supplementary Table S2.

Across the 57 included studies, 9 were diagnostic accuracy studies [18,19,20,21,22,23,24,25,26], 19 were predictive or longitudinal [27,28,29,30,31,32,33,34,35,36,37,38,39,40,41,42,43,44,45], and 29 were descriptive or exploratory [9,13,39,46,47,48,49,50,51,52,53,54,55,56,57,58,59,60,61,62,63,64,65,66,67,68,69]. Only diagnostic studies contributed to pooled effect-size estimation, whereas predictive and descriptive studies were analyzed narratively. This stratification was applied to avoid conceptual ambiguity and ensure that meta-analytic inferences reflect actual diagnostic performance rather than developmental associations or feasibility outcomes.

Sample sizes ranged from 18 to 635 participants per study.

Geographically, the 57 studies were conducted across a diverse range of settings, with the largest number originating from high-income countries such as the United States, Sweden, Italy, Switzerland, the United Kingdom, Israel, China, and Japan. A smaller number of studies were carried out in lower-resource settings, including Peru and Malta, often with a focus on feasibility and algorithm development for scalable screening tools.

The included studies encompassed a total of 5214 participants (mean age range: 9–30 months).

Across the included studies, a wide variety of stimuli were employed: static image presentations (*n* = 22); dynamic social videos (*n* = 18); gaze-contingent paradigms (*n* = 10); live, naturalistic interactions (*n* = 7).

Eye-tracking hardware included Tobii (*n* = 31), SMI (*n* = 12), and other infrared-based systems. Stimuli calibration procedures were standardized in 85% of studies.

Given the methodological diversity across included studies, we report findings in three complementary subsections: (1) behavioral eye-tracking and pupillometry results derived from traditional experimental paradigms, (2) machine learning-based classification approaches, and (3) quantitative meta-analysis. This structure enables each type of evidence to be interpreted within its appropriate methodological context.

### 3.3. Main Behavioral Findings

A total of 51 studies contributed to the behavioral synthesis [9,18,19,20,21,22,23,24,25,26,27,28,29,30,31,32,33,34,35,36,37,39,40,41,42,43,44,45,46,47,48,49,50,51,53,55,57,59,60,61,62,63,64,65,66,67,68,69,70,71,72]. These studies examined group-level differences in gaze behavior, pupillometry, social attention, and attentional dynamics.

Across the included studies, eye-tracking paradigms varied in complexity, but several consistent patterns emerged. Most studies converged on differences in social attention allocation, including reduced gaze to faces and atypical orienting toward social cues in children later diagnosed with ASD. Studies using pupillometry reported altered autonomic arousal and slower pupillary light reflex responses, while dynamic gaze-contingent paradigms highlighted atypical processing of interactive social stimuli. Despite methodological diversity, the findings consistently indicated that early differences in visual attention—particularly those related to social processing—were detectable across age ranges, from early infancy to preschool years.

### 3.4. Conceptual Themes

Across the included literature, several conceptual themes emerged that characterize how eye-tracking and pupillometry differentiate children with ASD from typically developing peers.

(1) Social attention differences as a core feature. A consistent finding across paradigms was reduced attention to socially relevant cues—faces, mutual gaze, biological motion, and joint attention signals. This pattern appeared robust across age groups, including infants at elevated familial likelihood and children with confirmed ASD diagnoses. Studies varied in design but converged on the conclusion that attenuated social orienting represents a stable marker across developmental stages.
(2) Altered autonomic and sensory responsivity. Pupillometry-based studies highlighted atypical modulation of autonomic arousal, particularly slower or blunted pupillary light reflex responses and elevated baseline pupil size in some cohorts. These findings are showing broader differences in sensory responsivity that accompany social attention atypicalities, reflecting potential dysregulation in underlying neurophysiological systems.
(3) Differences in interactive and dynamic processing. Paradigms involving live or gaze-contingent interaction revealed inconsistencies in responsiveness to contingent social cues. Children with ASD or infants who later developed ASD demonstrated reduced adaptation to shifting gaze cues and atypical modulation of attention during dynamic, socially meaningful exchanges.
(4) Developmental trajectories rather than static differences. Longitudinal studies consistently showed that atypicalities in gaze behavior are detectable early, may widen over time, and correlate with later diagnostic outcomes or developmental functioning. These developmental data underscore the value of eye-tracking as a tool not only for characterizing differences but also for tracing their evolution across infancy and early childhood.

Together, these themes emphasize convergent mechanisms—differences in social attention, autonomic responsivity, and dynamic processing—rather than isolated study results, providing a clearer conceptual understanding of how eye-tracking contributes to early detection of ASD.

### 3.5. Machine Learning Applications

The ML synthesis included 6 studies that used computational models to classify ASD or predict developmental outcomes based on eye-tracking features [21,28,41,42,47,50]. Classification accuracy improved substantially with a sensitivity: 78–89% and a specificity: 74–86%. Composite indices, such as the Autism Risk Index (ARI), demonstrated strong correlations with ADOS-2 scores (r > 0.75), highlighting the potential of algorithmic approaches for individualized risk stratification.

### 3.6. Critical Appraisal of Machine Learning Studies

Several included studies applied machine learning models to eye-tracking data (*n* = 6). However, critical evaluation revealed substantial methodological heterogeneity and recurrent limitations.

First, training datasets were generally small, often comprising fewer than 50–100 participants, raising concerns regarding model overfitting and limited generalizability. Second, cross-validation procedures were inconsistently reported; many studies relied on within-sample validation without independent test sets, and only a minority used hold-out or external validation. Third, class imbalance was common, with disproportionate representation of ASD vs. non-ASD samples, yet few studies reported bias-mitigation strategies (e.g., resampling, weighting).

Furthermore, reporting of feature-selection procedures, hyperparameter tuning, and prevention of data leakage was inconsistent. In several cases, diagnostic labels were derived retrospectively or via proxy diagnostic indicators, which may introduce additional labeling bias.

Overall, although ML approaches are promising, the methodological limitations of existing studies restrict confidence in their diagnostic utility.

### 3.7. Meta-Analysis

**Figure 2:**
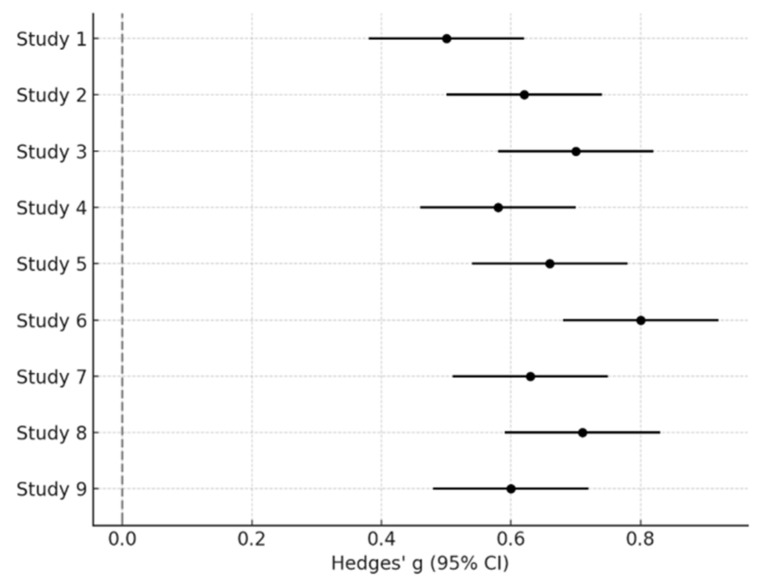
Forest Plot of social fixation time differences between ASD and TD infants.

The quantitative meta-analysis included 9 studies for which sufficient statistical information (means, SDs, or convertible estimates) was available [18,19,20,26,31,32,64,67,69]. A random-effects meta-analysis of social fixation time revealed a moderate-to-large group difference between infants with ASD and typically developing (TD) peers (Hedges’ g = 0.65; 95% CI: 0.48–0.82; *p* < 0.001), with moderate heterogeneity (I^2^ = 58%) (Figure 2).

In Figure 2, each study is represented by a square proportional to its weight in the random-effects model, with horizontal lines indicating the 95% confidence intervals. Studies positioned left of zero indicate reduced social fixation in ASD compared with TD peers. The diamond at the bottom represents the pooled effect size (Hedges’ g = 0.65), with its width corresponding to the overall confidence interval. The absence of confidence interval overlap across several studies visually supports the robustness of the group difference.

Sensitivity analyses excluding studies at high risk of bias yielded a similar pooled estimate (Hedges’ g = 0.61; 95% CI: 0.45–0.79), supporting the robustness of the result. Assessment of publication bias using funnel plot inspection and Egger’s regression did not indicate significant asymmetry (*p* = 0.27).

Although the pooled standardized mean difference indicates measurable group-level differences, effect sizes alone do not directly quantify diagnostic performance. Most studies did not report sensitivity, specificity, or decision thresholds; therefore, we interpreted effect magnitudes in light of typical accuracy trade-offs. Across diagnostic studies, effect sizes in the moderate range suggest that eye-tracking measures may distinguish ASD from TD at the group level, but may not achieve clinically actionable sensitivity/specificity without additional decision rules, multimodal features, or validated thresholds. The absence of consistent reporting of ROC-based metrics limits direct estimation of diagnostic accuracy.

**Figure 3:**
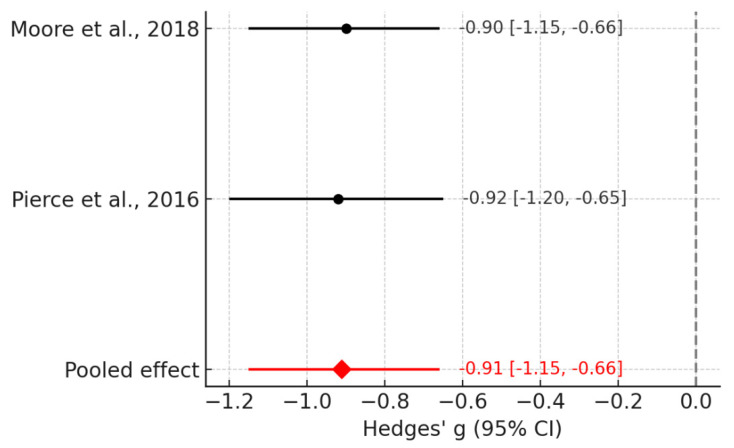
Forest plot for the Geo/Social paradigm meta-analysis [18,64].

In a more restricted synthesis focusing on the Geo/Social paradigm [18,64] children with ASD demonstrated significantly reduced attention to social stimuli compared with TD peers (Figure 3). The pooled random-effects effect size was large (Hedges’ g = −0.91; 95% CI: −1.15 to −0.66; *p* < 0.0001), with negligible between-study heterogeneity (I^2^ = 0%). These findings highlight reduced social fixation in ASD as a robust candidate biomarker detectable with eye-tracking.

In Figure 3, focusing on the Geo/Social paradigm, both included studies demonstrate large negative effect sizes, indicating substantially reduced attention to social stimuli among children with ASD. The lack of between-study heterogeneity (I^2^ = 0%) is visually evident from the near-identical positions of the study estimates and the narrow pooled-effect diamond.

By contrast, studies using other paradigms were too heterogeneous for quantitative pooling. Joint attention tasks frequently reported reduced gaze alternations and less consistent following of others’ gaze in infants at elevated likelihood for ASD, although effect sizes varied across contexts. Live interaction paradigms, including face-to-face eye-tracking, similarly indicated reduced attention to faces and diminished responsiveness to social gaze in ASD, but methodological variability limited comparability. Pupillary response studies reported atypical autonomic reactivity, with some showing hypersensitive light reflexes and others blunted constriction or dilation, yielding inconsistent associations with later diagnosis.

Taken together, the evidence suggests consistent alterations in early social attention and autonomic responses in ASD, but standardization of paradigms and outcome measures will be essential to strengthen their utility as biomarkers.

### 3.8. Differences Between High-Risk Infants and Confirmed ASD Groups

Across the included literature, study populations varied substantially with regard to diagnostic certainty. Among the 57 studies, 9 involved children with confirmed ASD diagnoses, whereas 19 predictive/longitudinal studies relied on high-likelihood (familial-risk or elevated-risk) infant cohorts without confirmed diagnostic outcomes at the time of testing. An additional 29 exploratory studies included mixed or unspecified samples.

This distinction was systematically considered during synthesis. Only studies involving confirmed ASD vs. typically developing controls were included in the meta-analysis. In contrast, studies involving high-risk infants were synthesized separately because early familial-risk status does not equate to later ASD diagnosis and reflects a fundamentally different question (prediction vs. diagnostic performance).

Across high-risk infant cohorts, gaze differences were often smaller, more variable, or developmentally unstable, consistent with the expectation that not all high-risk infants progress to ASD. In contrast, studies comparing confirmed ASD vs. TD groups showed more consistent reductions in social fixation, albeit with methodological heterogeneity. These patterns underscore the necessity of interpreting high-risk and confirmed-ASD findings as addressing distinct developmental and clinical constructs.

### 3.9. Risk of Bias

Using QUADAS-2, each study was evaluated across four domains: Patient Selection, Index Test, Reference Standard, and Flow/Timing. The overall judgment of risk of bias was categorized as Low, High, or Unclear. A summary of individual study characteristics is provided in Supplementary Table S3.

Most of the included studies demonstrated a Low risk of bias across the QUADAS-2 domains. Patient Selection and Index Test were consistently rated as Low risk, reflecting appropriate recruitment strategies and the use of standardized, validated eye-tracking paradigms. The Reference Standard was often a confirmed clinical ASD diagnosis based on gold-standard tools such as ADOS and ADI-R, which minimized bias in diagnostic ascertainment.

Several studies were rated as Unclear in the Reference Standard domain because they relied on proxy measures of autism likelihood (e.g., familial risk status) or developmental outcomes, rather than confirmed ASD diagnoses. This was particularly common in infant sibling studies or feasibility studies where diagnostic outcomes were not yet available.

Flow/Timing raised some concerns in studies that were strictly cross-sectional or feasibility-focused. These designs provided valuable preliminary data but limited conclusions about longitudinal predictive value.

Only one study [54] was rated as High risk overall. This was due to its very small sample size (N = 8 ASD participants) and reliance on an unvalidated prototype algorithm, which introduced concerns in both Patient Selection and Index Test domains.

Overall methodological quality was high across most included studies. As shown in Supplementary Table S3, the majority of studies (over 85%) were judged to have low risk of bias in the Index Test and Flow/Timing domains. Higher variability was observed in the Reference Standard domain, particularly in studies involving infant sibling high-likelihood cohorts, where diagnostic outcomes were not yet available at the time of publication. A small number of studies demonstrated high risk of bias in Patient Selection or Index Test due to reliance on public image datasets or early-stage algorithm development. No study was excluded based on quality assessment.

## 4. Discussion

The present meta-analysis reveals a moderate to large group difference in social fixation between infants with ASD and TD peers (Hedges’ g = 0.65). This effect closely aligns with a previous meta-analysis [10] of ET and social attention (g = 0.55) reinforcing reduced social attention as a reproducible early feature of ASD. Such convergence indicates that infants and toddlers with or at elevated likelihood for ASD consistently allocate less visual attention to social stimuli, reflecting difficulties in prioritizing socially salient information.

However, moderate heterogeneity (I^2^ = 58%) suggests that gaze differences vary systematically with task characteristics. Our most consistent findings emerged when we restricted our analysis to the Geo/Social paradigm [18,64], which contrasts social and geometric stimuli under standardized conditions. Focusing on this paradigm, heterogeneity dropped to 0%, indicating that task characteristics substantially modulate observed effect sizes.

Previous meta-analytic work [10] reported comparable heterogeneity and attributed the effect-size variability to the social content of the stimuli, larger effects emerging in scenes with higher social content (more than one-person, higher level of activity). The Geo/Social paradigm, in contrast, isolates the motivational dimension of attention by directly contrasting social and non-social (geometric) input. The consistent preference for geometric content among infants diagnosed with ASD parallels findings that early variations in social–visual engagement predict later diagnostic outcomes [4]. Such early biases could reduce infants’ opportunities to extract socially adaptive information and to develop typically joint-attention trajectories [27].

Beyond overall social fixation differences, qualitative analyses revealed convergent abnormalities across multiple attentional subsystems. Infants and toddlers with or at elevated likelihood for ASD frequently showed reduced gaze following [27,30], a core component of joint attention, compared with TD peers [73]. They also exhibited slower attentional shifts towards social cues and reduced focus on the eye region [19,20,28], consistent with reduced salience of socially informative features.

At the oculomotor level, findings suggest that gaze untypicalities, such as increased randomness and variability in saccadic patterns, are not explained by primary oculomotor deficits, as basic saccadic parameters appear intact, but rather reflect noisier top–down regulation of visual exploration [22,32]. Pupillary measures show a similar pattern of dysregulation, while some studies describe hypersensitive light reflexes in infants with a high risk of ASD [45], others report reduced dilation to social stimuli [33]. These results indicate that while the motor component of the gaze is preserved, the dynamic regulation of arousal and attention is less adapted to respond to socially meaningful input.

The present synthesis also highlights the translational potential of algorithmic approaches to aid early ASD diagnosis. Machine learning algorithms trained on gaze metrics achieved high diagnostic accuracy (sensitivity: 78–89%, specificity: 74–86%), while composite indices such as the Autism Risk Index (ARI) [3], showed robust correlations with ADOS-2 scores (r > 0.75), supporting the validity of data-driven modeling in individualized risk estimation. These findings underscore the added value of ET as an early adjunctive screening tool for ASD, detectable within the first year of life. Because it can be implemented in less than 10 min, ET is feasible for large-scale screening. The integration of AI/ML enhances diagnostic performance and may facilitate individualized risk stratification.

While most studies (over 85%) were rated as low risk across several QUADAS-2 domains, important limitations emerged. A substantial proportion of studies relied on small sample sizes, particularly in high-likelihood infant cohorts, which reduces statistical power and increases uncertainty around effect estimates. Several early-phase or feasibility studies employed unvalidated or prototype eye-tracking paradigms, limiting the comparability of outcomes across studies. In addition, a notable number of infant sibling or high-likelihood cohorts used proxy diagnostic status (e.g., elevated familial risk or developmental screening outcomes) rather than confirmed ASD diagnoses at follow-up, resulting in an unclear risk of bias in the Reference Standard domain. These studies provide valuable longitudinal insight but reduce the certainty of diagnostic accuracy in very early infancy. Finally, methodological variability—such as differences in calibration procedures, stimuli design, and definition of areas of interest—introduced heterogeneity in the Index Test domain even when risk was formally rated as low. Overall, the evidence base for eye-tracking as a diagnostic or predictive tool in autism research is robust, with most studies meeting high standards of quality but caution is warranted when interpreting pooled estimates, particularly for paradigms with limited validation.

Several limitations should be considered when interpreting these findings. First, the lack of standardization across eye-tracking paradigms and stimuli limits cross-study comparability. Second, considerable variability in sample sizes and participant characteristics may have reduced the generalizability of effect estimates. In addition, practical barriers such as hardware costs, calibration challenges, and the need for trained personnel currently constrain large-scale implementation in clinical or community settings.

Another important methodological consideration is the heterogeneity of study types. To avoid conceptual ambiguity, we explicitly distinguished diagnostic from predictive and descriptive studies. Only the 9 diagnostic accuracy studies were pooled meta-analytically. The remaining 19 predictive and 29 descriptive studies addressed developmental associations, feasibility, or paradigm exploration and therefore could not be meaningfully combined with diagnostic outcomes. This distinction reinforces the interpretability of our findings and avoids overstating diagnostic utility based on studies that did not directly evaluate diagnostic performance.

An important conceptual distinction in the literature relates to high-risk infants versus children with confirmed ASD diagnoses. These groups are not interchangeable. High-risk infants represent a heterogeneous population, and only a subset eventually receive an ASD diagnosis. Consequently, their gaze patterns reflect probabilistic developmental trajectories, not established clinical conditions. In contrast, findings from confirmed ASD samples more directly reflect diagnostic differences.

To address this, our review explicitly separated these populations: only confirmed-ASD comparisons contributed to the meta-analysis, while high-risk cohorts were synthesized narratively. This approach highlights that inconsistent findings in early infancy should not be interpreted as evidence against diagnostic value but rather as reflecting developmental variability and uncertainty inherent to high-risk samples.

Although ML approaches appear promising for augmenting early ASD identification, the current evidence base is limited by small training sets, lack of external validation, and inconsistent reporting of methodological safeguards (e.g., feature selection, cross-validation, and prevention of data leakage). These issues increase the risk of overfitting and optimistic bias, particularly when diagnostic labels are based on proxy measures or when class imbalance is not addressed. As a result, ML-based findings should be interpreted with caution. Robust multicenter datasets, standardized pipelines, and transparent reporting following established ML reporting guidelines (e.g., TRIPOD-ML) are needed before clinical implementation can be considered.

Although our meta-analysis quantified group differences through effect sizes, standardized mean differences cannot be directly interpreted as diagnostic accuracy. Very few studies reported sensitivity, specificity, AUC, or explicit decision thresholds, which are essential to evaluating clinical performance. As a result, the diagnostic implications of the observed effect sizes must be considered cautiously. Medium-sized effects may correspond to modest diagnostic accuracy, especially when sensitivity–specificity trade-offs are considered. Future research should adopt diagnostic accuracy standards—including ROC analyses, calibration curves, external validation, and transparent threshold reporting—to support clinically meaningful interpretation.

Future research should therefore prioritize the development of standardized and validated ET tasks, the coordination of large multi-center longitudinal cohorts, and the integration of gaze-based metrics with complementary biomarkers (e.g., genetic, neuroimaging, and behavioral data) to refine individualized risk prediction models for ASD.

## 5. Conclusions

This systematic review and meta-analysis examined the current evidence supporting eye-tracking-based measures as early markers of ASD. We identified a moderate-to-large reduction in social fixation, with more consistent effects observed with standardized task paradigms. We also summarize the reported performance of ML approaches while noting important methodological constraints. Standardization and clinical validation are essential next steps before considering transition of ET from research to practice.

## Data Availability

No new data were created or analyzed in this study. Data sharing is not applicable to this article.

## Supplementary Materials

The following supporting information can be downloaded at: https://www.mdpi.com/article/10.3390/jcm14248801/s1. Supplementary Table S1—Full database search strategies; Supplementary Table S2: Characteristics of included studies; Supplementary Table S3—QUADAS-2 quality assessment table; Supplementary Table S4—Full list of excluded full-text studies. Reference [74] is cited in the supplementary materials.

## Abbreviations

The following abbreviations are used in this manuscript:

ASD	Autism Spectrum Disorder
ADOS	Autism Diagnostic Observation Schedule
ADI-R	Autism Diagnostic Interview-Revised
ET	Eye-tracking
TD	typically developing
AI	artificial intelligence
ML	machine learning
QADAS2	Quality Assessment of Diagnostic Accuracy Studies 2
ARI	Autism Risk Index
