# Supplementary material for: Eye-Tracking as a Screening Tool in the Early Diagnosis of Autism Spectrum Disorder: A Systematic Review and Meta-Analysis
Source: J Clin Med. 2025 Dec 12;14(24):8801. doi: 10.3390/jcm14248801 (PMC12733574; doi:10.3390/jcm14248801)
Supplement: Supplementary file 1 [file jcm-14-08801-s001.zip › Supplementary Table S1.pdf]

Supplementary Table S1 - Full database search strategies

Database	Search String	Filters/Notes
**PubMed**	("Eye Movement Measurements"[MeSH Terms] OR "eye tracking"[Title/Abstract] OR "gaze behavior"[Title/Abstract]) AND ("Autism Spectrum Disorder"[MeSH Terms] OR "autism"[Title/Abstract] OR "ASD"[Title/Abstract]) AND ("Early Diagnosis"[MeSH Terms] OR "early diagnosis"[Title/Abstract] OR "early detection"[Title/Abstract] OR "infants"[Title/Abstract] OR "toddlers"[Title/Abstract]) AND ("2015/01/01"[Date - Publication] : "2025/07/28"[Date - Publication])	Language: English; Humans
**Scopus**	(TITLE-ABS-KEY("eye tracking") OR TITLE-ABS-KEY("eye-tracking") OR TITLE-ABS-KEY("gaze behavior") OR TITLE-ABS-KEY("eye movement")) AND (TITLE-ABS-KEY("autism spectrum disorder") OR TITLE-ABS-KEY("autism") OR TITLE-ABS-KEY("ASD")) AND (TITLE-ABS-KEY("early diagnosis") OR TITLE-ABS-KEY("early detection") OR TITLE-ABS-KEY("early identification") OR TITLE-ABS-KEY("infants") OR TITLE-ABS-KEY("toddlers")) AND PUBYEAR > 2014 AND PUBYEAR < 2026	Language: English
**Web of Science (Core Collection)**	TS=("eye tracking" OR "eye-tracking" OR "gaze behavior" OR "eye movement") AND TS=("autism spectrum disorder" OR autism OR ASD) AND TS=("early diagnosis" OR "early detection" OR "early identification" OR infants OR toddlers)	Document types: Article; Review excluded
**Medline (via Ovid)**	("eye tracking" OR "gaze behavior" OR "eye movement") AND ("autism spectrum disorder" OR autism OR ASD) AND ("early diagnosis" OR "early detection" OR "early identification" OR infants OR toddlers)	English language; Human subjects
**Cochrane Library**	(eye tracking OR eye-tracking OR gaze behavior OR eye movement) AND (autism spectrum disorder OR autism OR ASD) AND ("early diagnosis" OR "early detection" OR "early identification" OR infants OR toddlers)	Search in Cochrane Reviews, CENTRAL Trials
