# Supplementary material for: Eye-Tracking as a Screening Tool in the Early Diagnosis of Autism Spectrum Disorder: A Systematic Review and Meta-Analysis
Source: J Clin Med. 2025 Dec 12;14(24):8801. doi: 10.3390/jcm14248801 (PMC12733574; doi:10.3390/jcm14248801)
Supplement: Supplementary file 1 [file jcm-14-08801-s001.zip › Supplementary Table S2 final.pdf]

Supplementary Table S2: Characteristics of included studies

#	First author, Year	Country	Study design	Sample size (ASD / TD / HR)	Age range (months)	Stimuli type	Eye-tracking paradigm	Main outcomes	Key findings
1	Pierce, 2016	USA	Cross-sectional	37 / 22 / NR	12–36	Geometric vs. social images	Static geometric vs social images	Fixation duration; preference scores	ASD group showed visual preference for geometric images; linked to symptom severity.
2	Nyström, 2019	Sweden	Longitudinal	NR	6–18	Social interaction cues	Joint-attention videos / live tasks	Gaze shifts; joint attention metrics	Reduced joint attention/gaze following predicted later ASD emergence.
3	Robain, 2022	Switzerland	Cross-sectional	NR	36–60 (preschoolers)	Cartoons	Cartoon social stimuli	Face & eye fixation	Reduced social orienting in ASD preschoolers.
4	Hata, 2025	Japan	Longitudinal	NR	3–12	Talking faces	Talking face stimuli	Mouth vs eye fixation	Emerging sensitivity to talking mouth in

#	First author, Year	Country	Study design	Sample size (ASD / TD / HR)	Age range (months)	Stimuli type	Eye-tracking paradigm	Main outcomes	Key findings
									HR and low-likelihood infants.
5	Kwon, 2019	USA	Cross-sectional	NR	24–36	Multiple contexts (face/eye region)	Face stimuli across contexts	Eye-region fixation	Reported typical levels of eye-region fixation in ASD toddlers across contexts.
6	Gliga, 2015	UK	Longitudinal (BASIS)	NR	9–18	Visual search arrays	Visual search tasks	Visual search efficiency	Enhanced visual search in infancy predicted emerging autism symptoms.
7	Nele et al., 2015	NR	Cross-sectional	NR	~5	Familiar face; direct gaze	Familiar face / direct gaze	Attention allocation	Social info processing differences in infants at risk (5 months).

#	First author, Year	Country	Study design	Sample size (ASD / TD / HR)	Age range (months)	Stimuli type	Eye-tracking paradigm	Main outcomes	Key findings
8	X. Wang et al., 2020	NR	Cross-sectional	NR	NR	Social vs non-social under competition	Social stimuli under competitive conditions	Orientation / processing metrics	Orientation to social stimuli studied; details NR.
9	Thorup, 2024	NR	Observational	NR	Infant age range NR	Live interaction	Parent–infant interaction (face looking)	Frequency of face looking	Less frequent face looking related to autism likelihood status (but not diagnosis).
10	Bradshaw, 2023	NR	Feasibility study	NR	Early infancy	Naturalistic scenes	Head-mounted/home-based ET	Feasibility metrics	Home-based head-mounted ET feasible in early infancy.
11	Wang, Q., et al., 2018	USA	Cross-sectional / methods	NR	Toddlers	Faces (cohesion-based analysis)	Cohesion-based gaze metrics	Atypical gaze operationalization	Proposed cohesion-based approach to

#	First author, Year	Country	Study design	Sample size (ASD / TD / HR)	Age range (months)	Stimuli type	Eye-tracking paradigm	Main outcomes	Key findings
									quantify atypical gaze.
12	Costanzo, 2025 (Appl. Sci.)	Italy	Single-center longitudinal	NR	12 → 24 (12 & 24)	NR in draft	Standard ET at 12 months	Joint attention; socio-communicative outcomes	Altered visual attention at 12 months predicted joint attention & outcomes at 24 months.
13	Frazier, 2021	Multi-country	Cross-sectional	NR	Infants/toddlers NR	Social attention tasks	Cross-cultural ET tasks	Social attention metrics	Social attention as cross-cultural transdiagnostic risk marker.
14	Sacrey, 2023	NR	Comparative study	NR	Toddlers	Live vs on-screen tasks	Face-to-face vs computer-based tasks	Attention task performance	Compared strengths/challenges across modalities in ASD vs NT.

#	First author, Year	Country	Study design	Sample size (ASD / TD / HR)	Age range (months)	Stimuli type	Eye-tracking paradigm	Main outcomes	Key findings
15	Ziv, 2024	Israel	Cross-sectional	NR	Children (age NR)	NR in draft	Oculomotor analysis	Oculomotor randomness	Higher oculomotor randomness in autistic children; correlated with severity.
16	Avni, 2021	Israel	Cross-sectional	NR	Young children	Basic oculomotor tasks	Basic oculomotor tests	Oculomotor function	Basic oculomotor function similar in ASD and TD young children.
17	Muratori, 2019	Italy	Longitudinal / ET	NR	Toddlers	Faces and objects	Face & object attention over time	Fixation changes over time	Attention to faces/objects changed over time in ASD toddlers.
18	Masedu, 2021	Italy	Cross-sectional	NR	Toddlers	NR in draft	Visual fixation trajectories	Fixation transition metrics	Compared fixation trajectories

#	First author, Year	Country	Study design	Sample size (ASD / TD / HR)	Age range (months)	Stimuli type	Eye-tracking paradigm	Main outcomes	Key findings
							(Markov model)		between ASD and TD toddlers.
19	Fish et al., 2021	UK	Longitudinal (BASIS/STAARS)	NR	9–24	Light reflex paradigm	Pupillary light reflex & ET	Pupillary development; ASD association	Pupillary reflex associated with ASD genetic liability and later diagnosis.
20	Camero, 2021	Spain?	Cross-sectional	NR	Toddlers	Gaze following; pupil dilation	Gaze following & pupil dilation	Gaze-following, pupil metrics	Both gaze-following and pupil dilation investigated as early markers.
21	Jaradat, 2024	Jordan?	Methods/ML study	NR	NR	NR in draft	Machine learning on ET	Classification performance	ML applied to ET for ASD diagnosis (Diagnostics).

#	First author, Year	Country	Study design	Sample size (ASD / TD / HR)	Age range (months)	Stimuli type	Eye-tracking paradigm	Main outcomes	Key findings
22	Kojovic, 2024	Switzerland	Developmental dynamics	NR	Infant/toddler range NR	Social interactions	Visual exploration of social interactions (ET)	Developmental dynamics of visual exploration in ASD.	
23	Keemink, 2019	Australia	Experimental	NR	Infants	Expressive faces	Gaze-contingent expressive faces	Eye movements; behavioural responses	Responses to interactive gaze-contingent faces in infants and infant siblings.
24	Vernetti, 2024	USA	Feasibility / live ET	NR	Toddlers	Face-to-face ET	Face-to-face live ET	Feasibility metrics; impact of familiarity/face covering	Live face-to-face ET feasible; familiarity/covering affect measures.
25	Vargas-Cuentas, 2017	Peru	Algorithm development	NR	Children	NR in draft	Algorithm for ET	Algorithm validation	Developed ET algorithm as potential early diagnostic tool in

#	First author, Year	Country	Study design	Sample size (ASD / TD / HR)	Age range (months)	Stimuli type	Eye-tracking paradigm	Main outcomes	Key findings
									low-resource setting.
26	Franchini, 2017	Switzerland	Cross-sectional	NR	Preschoolers	Social orienting; joint attention	Social orienting & joint attention	Social orienting metrics	Social orienting and joint attention in preschoolers with ASD.
27	Sun, 2024	China?	Multimodal (EEG+ET)	NR	Early ages NR	Interest paradigm stimuli	Interest paradigm with EEG+ET	Combined EEG & ET outcomes	Combined measures for early identification of ASD.
28	Meng, 2023	China	ML study	NR	Children	Real vs artificial faces	Real & artificial face scanning	ML classification metrics	ML-based early diagnosis using eye movements across face types.

#	First author, Year	Country	Study design	Sample size (ASD / TD / HR)	Age range (months)	Stimuli type	Eye-tracking paradigm	Main outcomes	Key findings
29	Tarrit, 2023	USA	Cross-age study	NR	Children & adults	Saccadic adaptation task	Saccadic adaptation tasks	Saccadic adaptation	No differential saccadic adaptation in ASD vs controls.
30	Parsons, 2019	UK	Frontiers psychology	NR	Infants at familial risk	Gaze following; objects	Gaze following & attention to objects	Gaze-following metrics	Gaze following and object attention in infants at risk for ASD.
31	Wang R.K., 2024	NR	Methods/metrics	NR	Toddlers/preschool	Various (metric system proposal)	New ET metrics system	Value in early diagnosis	Proposed new metrics system for early ASD diagnosis.
32	Kong, 2022	China?	Cross-sectional	NR	Toddler & preschool	NR in draft	ET scanning patterns	Different ET patterns by age group	Different eye-tracking patterns in toddlers vs preschool children with ASD.

#	First author, Year	Country	Study design	Sample size (ASD / TD / HR)	Age range (months)	Stimuli type	Eye-tracking paradigm	Main outcomes	Key findings
33	Zeng, 2023	USA?	Longitudinal	NR	6–14 months	Social motivation context	Social motivation & gaze following	Social motivation predicts gaze following	Social motivation predicted gaze following between 6–14 months.
34	Jones et al., 2023	USA	Development & replication (JAMA Net Open)	NR	Infants/toddlers	Interactive faces	Social visual engagement metrics (objective)	Objective measures of social visual engagement for early diagnosis.	
35	Costanzo, 2022	Italy	Eye-tracking clinical study	NR	High-risk siblings	Live social engagement measures	Clinical ET	Insights from HR siblings without ASD	Examined HR siblings; provided clinical ET insights.
36	Keehn, 2024	USA	Primary care study	NR	Early ages NR	ET tasks	ET biomarkers in primary care	Feasibility/biomarkers	Investigated ET biomarkers and ASD diagnosis in primary care.

#	First author, Year	Country	Study design	Sample size (ASD / TD / HR)	Age range (months)	Stimuli type	Eye-tracking paradigm	Main outcomes	Key findings
37	Krogh-Jespersen, 2018	USA	Eye-tracking study	NR	24 (2-year-olds)	Biomarker ET in primary care	Goal prediction tasks	Goal prediction metrics	Goal prediction compared in 2-year-olds with and without ASD.
38	Lynch, 2018	USA	Adolescents (pupil study)	NR	Adolescents (not infants)	Goal prediction scenes	Pupillary response	Latency to constriction	Pupillary latency discriminated ASD from TD in adolescents.
39	Wang et al., 2025	NR	Autism Research	NR	Infants at high likelihood	Pupillary response	Face scanning; orienting to eyes	Orienting to/away from eyes	Studied orienting to/away from eyes in high-likelihood infants.
40	Rudling, 2024	Sweden	Live ET study	NR	Infants	Face scanning (eyes)	Live direct gaze responses	Associations to autism	Infant responses to direct gaze associated with autism metrics.

#	First author, Year	Country	Study design	Sample size (ASD / TD / HR)	Age range (months)	Stimuli type	Eye-tracking paradigm	Main outcomes	Key findings
41	Thorup et al., 2018	Denmark/Sweden	Longitudinal	NR	Infants	Direct gaze responses	Alternating gaze during social interaction	Alternating gaze frequency	Reduced alternating gaze associated with elevated later autism symptoms.
42	Nyström et al., 2017	Sweden	Experimental	NR	Infants at risk	Alternating gaze during social interaction	Responses to direct gaze	Short-timescale gaze alterations	Alterations in gaze behavior in at-risk infants occur on short timescales.
43	Jensen et al., 2021	Peru	Field/resource-limited study	NR	Young children	Responding to direct gaze	Gaze preference + M-CHAT-R	Screening combo	Combined gaze preference with M-CHAT-R for autism detection in resource-scarce setting.

#	First author, Year	Country	Study design	Sample size (ASD / TD / HR)	Age range (months)	Stimuli type	Eye-tracking paradigm	Main outcomes	Key findings
44	Fu et al., 2025	NR	Multi-method comparison	NR	Infant age NR	Gaze preference tasks	Multi-method ET comparisons	Social attention measures	Compared multiple methods for assessing infant social attention linked to ASD likelihood.
45	Nyström et al., 2015	Sweden	Longitudinal	NR	9–24	Various social attention tasks	Pupillary light reflex	Hypersensitive PLR	Hypersensitive pupillary light reflex in infants at risk for autism.
46	Moore et al., 2018	USA	Cross-sectional	NR	Early ages	Pupillary light reflex	Geometric preference subtype study	Geometric preference metrics	Identified consistent early-emerging geometric preference subtype in ASD.

#	First author, Year	Country	Study design	Sample size (ASD / TD / HR)	Age range (months)	Stimuli type	Eye-tracking paradigm	Main outcomes	Key findings
47	Yurkovic et al., 2021	USA	Head-mounted ET	NR	Children	Gaze following during live interaction	Naturalistic toy play (head-mounted ET)	Visual/manual exploration	Used head-mounted ET to study naturalistic exploration in ASD vs controls.
48	Chetcuti et al., 2024	Malta / Australia?	Feasibility	NR	Short protocol	Geometric vs social videos/images	2-minute ET protocol	Feasibility/identification	Demonstrated feasibility of a very short ET protocol to support early ID.
49	Yamashiro et al., 2019	USA	Cross-sectional	NR	Infants later diagnosed vs neurotypical	Naturalistic toy play	Primate face preferences	Shifting face preferences	Changing preferences for primate faces in infants later diagnosed with ASD.
50	Wass et al., 2015	UK	Longitudinal	NR	Infants (early)	Short ET protocol	Spontaneous fixation durations	Fixation length metrics	Shorter spontaneous fixation

#	First author, Year	Country	Study design	Sample size (ASD / TD / HR)	Age range (months)	Stimuli type	Eye-tracking paradigm	Main outcomes	Key findings
									durations in infants with later emerging autism.
51	Billeci et al., 2016	Italy	Cross-sectional	NR	Toddlers	Primate faces	Joint attention initiation vs response	Joint attention metrics	Disentangled initiation from response in joint attention tasks.
52	Wagner et al., 2020	USA	Emotional face attention	NR	Infants at high risk	Spontaneous fixation	Fearful face bias	Attentional bias metrics	Attentional bias to fearful faces in high-risk infants.
53	Kaliukhovich et al., 2021	NR	Cross-age	NR	Children & adults	Gaze following conditions	Biological motion preference	Visual preference for biological motion	Studied visual preference for biological motion in ASD.

#	First author, Year	Country	Study design	Sample size (ASD / TD / HR)	Age range (months)	Stimuli type	Eye-tracking paradigm	Main outcomes	Key findings
54	Zhao et al., 2021	China?	ML on face-to-face ET	NR	Children	JA tasks	Face-to-face conversational ET	Classification performance	ML classification using ET from face-to-face conversations.
55	Wei et al., 2023	NR	Systematic review/meta-analysis	NR	NR	Social vs non-social images	ET + ML review	Review outcomes	Systematic review on ML using eye-tracking to identify ASD. (included as background)
56	Viktorsson et al., 2024	NR	Observational	NR	18-month-olds	Children interacting	Viewing other children interacting	Timing of gaze allocation	Timing differences in how 18-month-olds with later autism view interactions.

#	First author, Year	Country	Study design	Sample size (ASD / TD / HR)	Age range (months)	Stimuli type	Eye-tracking paradigm	Main outcomes	Key findings
57	Jones et al., 2023 (duplicate entry set)	USA	JAMA Network Open (dev & replication)	NR	Infants/toddlers	Fearful faces	Objective social visual engagement	Objective measurement development	Developed and replicated objective measures to aid early diagnosis.

Abbreviations: ASD = Autism Spectrum Disorder; TD = Typically Developing controls; HR = High-Risk infants; NR = Not reported.
