# Supplementary material for: Eye-Tracking as a Screening Tool in the Early Diagnosis of Autism Spectrum Disorder: A Systematic Review and Meta-Analysis
Source: J Clin Med. 2025 Dec 12;14(24):8801. doi: 10.3390/jcm14248801 (PMC12733574; doi:10.3390/jcm14248801)
Supplement: Supplementary file 1 [file jcm-14-08801-s001.zip › Supplementary Table S3 final2.pdf]

Supplementary Table S3 - QUADAS-2 quality assessment table

Study (year, journal)	Patient Selection	Index Test	Reference Standard	Flow/Timing	Overall Conclusion
Avni et al., 2021, Autism Research	Low risk (young children, ASD vs TD, clear criteria)	Low risk (oculomotor saccade/fixation measures)	Low risk (clinical ASD diagnosis)	Low risk (cross-sectional design, adequate controls)	Low
Billeci et al., 2016, Transl Psychiatry	Low risk (toddlers ASD vs TD, small N)	Low risk (joint attention tasks)	Low risk (DSM-5 + ADOS-2 diagnosis)	Low risk (cross-sectional, standardized)	Low
Bradshaw et al., 2023, Dev Cogn Neurosci	Low risk (infants with and without ASD likelihood)	Low risk (head-mounted eye-tracking at home)	Some concern (feasibility study, not diagnostic outcome)	Some concern (short-term, feasibility focus)	Unclear
Camero et al., 2021, Children	Low risk (small NT vs high-likelihood toddlers, paired design)	Low risk (eye-tracking: gaze following + pupil dilation)	Some concern (no confirmed clinical ASD diagnosis, only likelihood)	Some concern (cross-sectional, early age only)	Unclear
Chetcuti et al., 2024, Sci Rep	Low risk (54 infants referred for concern)	Low risk (Gazefinder 2-min standardized task)	Unclear (clinical features assessed, not always diagnosis)	Low risk (single assessment)	Unclear
Costanzo et al., 2022, J Pers Med	Low risk (high-risk siblings, ASD, TD toddlers)	Low risk (eye-tracking joint attention tasks)	Low risk (clinical ASD diagnosis)	Low risk (cross-sectional, consistent protocol)	Low
Costanzo et al., 2025, Applied Sciences	Low risk (infants at elevated vs reduced likelihood, clear inclusion)	Low risk (standardized disengagement paradigm at 12m)	Low risk (ASD diagnosis at 24m)	Low risk (longitudinal design, repeated measures)	Low
Dewaele et al., 2015, Res Autism Spectr Disord	Low risk (infants at familial risk vs low risk)	Low risk (visual paired comparison eye-tracking)	Low risk (group-based outcome, no clinical diagnosis at that age)	Some concern (very early age, indirect outcomes)	Low
Fish et al., 2021, J Child Psychol Psychiatry	Low risk (large infant cohort, family-risk enriched)	Low risk (longitudinal pupillary light reflex eye-tracking)	Low risk (ASD diagnosis at 3 years)	Low risk (multi-timepoint follow-up: 9–24m + 3y outcome)	Low

Franchini et al., 2017, PLoS ONE	Low risk (preschoolers with ASD vs TD, clear criteria)	Low risk (dynamic social vs geometric stimuli)	Low risk (standardized clinical/parent measures)	Low risk (well-controlled stimuli)	Low
Frazier et al., 2021, Autism Research	Low risk (large US + Qatari cohorts, ages 1–17, broad inclusion)	Low risk (remote eye-tracking across multiple paradigms)	Low risk (clinical ASD/DD diagnoses confirmed)	Low risk (cross-sectional but well powered)	Low
Fu et al., 2025, J Autism Dev Disord	Low risk (infants, high vs low familial likelihood)	Low risk (eye-tracking vs manual coding attention)	Unclear (likelihood grouping, no diagnosis)	Low risk (multi-timepoint: 3–24m)	Unclear
Gliga et al., 2015, Curr Biol	Low risk (infant sibling design, HR vs LR groups)	Low risk (eye-tracking visual search task)	Low risk (ADOS, AOSI follow-up)	Low risk (assessed at 9m, 15m, 24m)	Low
Hata et al., 2025, Infant Behav Dev	Low risk (prospective infant cohorts, EL vs LL groups)	Low risk (longitudinal eye-tracking across 6–24 months)	Low risk (later vocabulary measures, ASD likelihood)	Low risk (multiple repeated measures)	Low
Jaradat et al., 2024, Diagnostics	High risk (used public image/scanpath datasets rather than clinically recruited participants; limited representativeness)	High risk (machine-learning models trained on augmented image datasets; risk of overfitting and dataset bias; not prospectively validated in clinical cohorts)	Unclear (labels derived from public datasets; diagnostic ascertainment/clinical confirmation not always described in detail)	Low risk (retrospective dataset analyses with clearly reported preprocessing and modeling steps)	High
Jensen et al., 2021, J Autism Dev Disord	Low risk (children 3–8y, ASD vs TD, clear recruitment)	Low risk (gaze preference + M-CHAT-R)	Low risk (ADOS-II/ADI-R diagnosis)	Low risk (cross-sectional, consistent)	Low
Jones et al., 2023, JAMA Netw Open	Low risk (large consecutive enrollment, N=1089)	Low risk (validated eye-tracking biomarker)	Low risk (gold-standard clinical diagnosis, blinded)	Low risk (prospective, consistent procedures)	Low
Keehn et al., 2024, JAMA Netw Open	Low risk (community-referred children, N=146)	Low risk (composite eye-tracking biomarker battery)	Low risk (blinded expert clinical diagnosis)	Low risk (prospective, consistent flow)	Low
Keemink et al., 2019, Dev Psychol	Low risk (infants tested with interactive gaze-contingent setup)	Low risk (eye-tracking in novel paradigm)	Unclear (no formal ASD diagnosis)	Low risk (standardized timing, clear protocol)	Unclear
Keemink et al., 2021, Autism Research	Low risk (large sample of TD infants and infant siblings recruited; inclusion criteria clear)	Low risk (gaze-contingent interactive eye-tracking paradigm with concurrent behavioural coding)	Unclear (infant sibling status used as risk marker; diagnostic follow-up not available for all participants at time of publication)	Low risk (cross-sectional with careful timing and behavioral video coding)	Unclear

Kojovic et al., 2024, eLife	Low risk (well-characterized developmental cohorts with clear inclusion/exclusion; diverse sample)	Low risk (rigorous eye-tracking measures of visual exploration of social interactions; validated analytic pipelines)	Low risk (diagnostic outcomes and clinical measures used as reference where relevant)	Low risk (detailed longitudinal / cross-sectional design with clear timing and handling of missing data)	Low
Kong et al., 2022, Front Psychiatry	Low risk (toddlers and preschool children with ASD and non-ASD controls; inclusion criteria described)	Low risk (novel ET metrics system with thorough reporting of metrics and quality control)	Low risk (clinical ASD diagnosis confirmed via standard instruments)	Low risk (cross-sectional clinical sample with standardized testing session)	Low
Krogh-Jespersen et al., 2018, Autism Res	Low risk (2-year-olds with/without ASD, well-defined)	Low risk (goal-prediction eye-tracking paradigm)	Low risk (clinical ASD diagnosis)	Low risk (standardized setting, clear timing)	Low
Kwon et al., 2019, JAACAP	Low risk (large toddler cohorts with ASD and controls)	Low risk (standardized vignettes)	Low risk (diagnosis confirmed)	Low risk (cross-sectional + longitudinal subsample)	Low
Lynch et al., 2017, Autism Res	Low risk (adolescents ASD vs TD, small but defined)	Low risk (pupillary light reflex latency)	Low risk (clinical ASD diagnosis)	Low risk (consistent procedure)	Low
Masedu et al., 2022, Brain Sciences	Low risk (ASD vs TD toddlers, matched)	Low risk (Markov chain modeling of fixation trajectories)	Low risk (ASD clinical diagnosis)	Low risk (cross-sectional design, clear reporting)	Low
Meng et al., 2023, Front Neurosci	Low risk (large sample: 117 ASD, 44 TD)	Low risk (cartoon vs real face paradigm with ML)	Low risk (clinical evaluation confirmed)	Low risk (standardized protocol)	Low
Moore et al., 2018, Mol Autism	Low risk (227 toddlers, ASD/TD/DD)	Low risk (GeoPref eye-tracking test)	Low risk (ADOS, MSEL, VABS diagnosis)	Low risk (cross-sectional, consistent)	Low
Muratori et al., 2019, Brain Sciences	Low risk (ASD toddlers vs TD, age-matched)	Low risk (eye-tracking on IJA tasks)	Low risk (clinical ASD diagnosis at baseline)	Some concern (small N, short 6-month follow-up)	Low
Nyström et al., 2015, Mol Autism	Low risk (10-month-olds, high vs low risk siblings)	Low risk (pupillary light reflex with eye-tracking)	Unclear (risk groups, no diagnostic confirmation)	Low risk (single assessment, standardized)	Unclear
Nyström et al., 2017, J Autism Dev Disord	Low risk (10-month-olds, small sample, risk groups)	Low risk (live direct-gaze task)	Unclear (no diagnostic outcome yet)	Low risk (short, standardized task)	Unclear

Nyström et al., 2019, Biol Psychiatry	Low risk (infant sibling design, clear inclusion criteria)	Low risk (live eye-tracking of RJA/IJA)	Low risk (ASD diagnosis at 36 months)	Low risk (repeated measures at 10, 14, 18 months)	Low
Parsons et al., 2019, Front Psychol	Low risk (infants at familial risk + controls; longitudinal)	Low risk (gaze-following and word learning tasks)	Low risk (later clinical outcomes)	Low risk (structured stimuli, clear timing)	Low
Pierce et al., 2016, Biol Psychiatry	Low risk (large, clearly defined cohorts)	Low risk (standardized eye-tracking paradigm)	Low risk (clinical ASD diagnosis as reference)	Low risk (prospective, with test–retest reliability)	Low
Robain et al., 2022, J Psychiatr Res	Low risk (ASD preschoolers and TD controls, well matched)	Low risk (validated cartoon and realistic eye-tracking tasks)	Low risk (clinical diagnosis of ASD)	Some concern (cross-sectional only, no follow-up)	Low
Rudling et al., 2023, Autism	Low risk (high-likelihood infants + controls, N=169)	Low risk (live eye-tracking to direct gaze)	Unclear (ASD diagnosis only in subset)	Low risk (longitudinal, 10–18 months)	Unclear
Sacrey et al., 2023, Autism Research	Low risk (ASD vs NT toddlers, matched age/sex)	Low risk (face-to-face + computer-based attention tasks)	Low risk (ASD diagnosis confirmed)	Low risk (cross-sectional only, clear protocol)	Low
Sun et al., 2024, Front Neurosci	Low risk (toddlers with ASD vs controls, defined groups)	Low risk (combined eye-tracking + EEG)	Low risk (standard clinical evaluation)	Low risk (structured protocol)	Low
Tarrit et al., 2023, Front Integr Neurosci	Low risk (ASD and TD groups well described)	Low risk (saccadic adaptation paradigm)	Low risk (clinical diagnosis referenced)	Low risk (clear adaptation protocol)	Low
Thorup et al., 2016, Mol Autism	Low risk (infants 10m, high vs low risk)	Low risk (gaze-following eyes/head cues)	Unclear (risk grouping, no diagnostic outcome)	Low risk (standardized procedure)	Unclear
Thorup et al., 2018, J Abnorm Child Psychol	Low risk (10-month-olds, high vs low risk)	Low risk (live gaze alternation task)	Unclear (ASD symptoms at 18m, not diagnosis)	Low risk (timed at 10 & 18m)	Unclear
Thorup et al., 2021, Autism	Low risk (infants at elevated likelihood vs TD, N≈126)	Low risk (live gaze-following tasks)	Low risk (clinical diagnosis at 3y)	Low risk (prospective, multi-timepoints)	Low

Thorup et al., 2024, Res Autism Spectr Disord	Low risk (parent–infant free play, EL vs LL infants)	Low risk (video-coded gaze shifts)	Some concern (no strong clinical reference, diagnosis not always linked)	Some concern (naturalistic setting, less controlled)	Unclear
Vacas et al., 2021, PLOS ONE	Low risk (ASD vs TD children, N=36, 41–73m)	Low risk (paired preference: faces vs objects)	Low risk (clinical diagnosis confirmed)	Low risk (cross-sectional, single session)	Low
Vargas-Cuentas et al., 2017, PLoS ONE	High risk (very small, only 8 ASD children)	High risk (prototype algorithm, unvalidated)	Unclear (clinical ASD diagnosis referenced but not detailed)	Low risk (brief, randomized video task)	High
Vernetti et al., 2024, Autism Research	Low risk (toddlers with ASD, ATYP, and TD recruited; diverse sample)	Low risk (live eye-tracking during face-to-face interaction)	Low risk (diagnosis based on established ASD criteria)	Low risk (short, standardized procedure)	Low
Viktorsson et al., 2024, J Autism Dev Disord	Low risk (98 toddlers, high/low likelihood)	Low risk (dynamic video of child interactions)	Low risk (ASD diagnostic outcome at 36m)	Low risk (prospective with outcome confirmation)	Low
Wagner et al., 2019, Emotion	Low risk (infant siblings, high vs low familial risk)	Low risk (emotional face disengagement)	Low risk (diagnosis at 36m)	Low risk (multi-timepoints 6–12m, prospective)	Low
Wang et al., 2018, Mol Autism	Low risk (large ASD, DD, TD toddler cohorts, clear inclusion)	Low risk (cohesion-based eye-tracking, validated conditions)	Low risk (clinical diagnosis and symptom severity)	Low risk (cross-sectional but robust sample sizes)	Low
Wang et al., 2020, Res Autism Spectr Disord	Low risk (ASD vs TD children, controlled groups)	Low risk (background search & paired preference tasks)	Low risk (clinical diagnosis)	Low risk (cross-sectional design, clear reporting)	Low
Wang et al., 2024, Front Psychiatry	Low risk (toddlers and preschoolers recruited clinically; groups described)	Low risk (new ET metrics system with detailed validation and reliability reporting)	Low risk (clinical diagnosis using standard instruments reported)	Low risk (single-session clinical testing with clear quality control)	Low
Wang et al., 2025, Autism Res	Low risk (infants at high likelihood vs TD)	Low risk (eye vs mouth orienting task)	Unclear (family risk grouping, no diagnosis)	Low risk (clear paradigm, timing described)	Unclear
Wass et al., 2015, Sci Rep	Low risk (high vs low risk infants, 6–9m)	Low risk (fixation durations, static images)	Low risk (ASD diagnosis at 36m)	Low risk (two cohorts, longitudinal)	Low
Yamashiro et al., 2019, Autism Res	Low risk (infants followed 6–18m)	Low risk (human vs monkey face tracking)	Low risk (diagnosis at 24m)	Low risk (repeated assessments)	Low

Yurkovic et al., 2021, Sci Rep	Low risk (ASD & TD 24–48m, matched groups)	Low risk (head-mounted eye-tracking in toy play)	Low risk (clinical diagnosis confirmed)	Low risk (single lab session, consistent)	Low
Zeng et al., 2023, Infancy	Low risk (82 infants tracked longitudinally; well-defined)	Low risk (multi-measure eye-tracking + pupillometry)	Unclear (developmental outcomes, not diagnosis)	Low risk (longitudinal, consistent follow-up)	Low
Ziv et al., 2024, Autism Research	Low risk (large ASD vs TD cohorts, ages 1–11)	Low risk (oculomotor randomness analysis from movie clips)	Low risk (ADOS-based ASD severity as reference)	Low risk (cross-sectional but large sample, repeated clips)	Low
