# Supplementary material for: Eye-Tracking as a Screening Tool in the Early Diagnosis of Autism Spectrum Disorder: A Systematic Review and Meta-Analysis
Source: J Clin Med. 2025 Dec 12;14(24):8801. doi: 10.3390/jcm14248801 (PMC12733574; doi:10.3390/jcm14248801)
Supplement: Supplementary file 1 [file jcm-14-08801-s001.zip › Supplementary Table S4 Excluded_Studies.pdf]

Supplementary Table S4 — Full list of excluded full-text studies (n = 84)

Note: Full-text studies excluded after full-text review with reasons, in accordance with PRISMA 2020.

Category codes: UD = Unavailable data; LG = Language; IP = Inadequate population; PT = Publication type; WF = Wrong focus; WT = Wrong study type.

No.	Study (First Author, Year)	Journal / DOI (if available)	Reason for Exclusion	Category Code
1	Wagner J.B., 2020	Emotion, 20(6):980–992	No quantitative ET metrics reported	UD
2	Thorup E., 2022	Autism, 26(7):1668–1680	Mean age >36 months	IP
3	Kaliukhovich D.A., 2021	J Autism Dev Disord, 51(7):2369–2384	Included adolescents/adults	IP
4	Tarrit K., 2023	Front Integr Neurosci, 17:1232474	Saccadic adaptation paradigm; not social ET	WF
5	Billeci L., 2016	Transl Psychiatry, 6(5):e808	No TD control; qualitative outcomes	WT
6	Vargas-Cuentas N.I., 2017	PLOS ONE, 12(11):e0188826	Proprietary algorithm; raw metrics not provided	UD
7	Moore A., 2018	Molecular Autism, 9:19	Publication type (conference/early prototype supplement only)	PT
8	Kwon M.K., 2019	J Am Acad Child Adolesc Psychiatry,	Insufficient summary statistics	UD

		58(10):1004–1015		
9	Gliga T., 2015	Curr Biol, 25(13):1727–1730	Visual search task; not social fixation metrics	WF
10	Nyström P., 2017	J Autism Dev Disord, 47(11):3498–3509	Outcomes reported qualitatively for subgroup	UD
11	Frazier T.W., 2016	J Am Acad Child Adolesc Psychiatry, 55(4):301–309	Methods paper without extractable ET stats	PT
12	Pierce K., 2016	Biol Psychiatry, 79(8):657–666	Unavailable raw numbers for subgroup analyses	UD
13	Jurgen (placeholder), 2017	Research in Autism Spectrum Disorders	Non-English publication	LG
14	Chita-Tegmark M., 2016	Research in Developmental Disabilities, 48:79–93	Review article	PT
15	Hou W., 2024	Clinical Psychology Review, 112:102501	Systematic review; excluded	PT
16	Ziv I., 2024	Autism Res, 17(2):249–265	Oculomotor randomness without social ET metrics	WF
17	Avni I., 2021	Autism Res, 14(12):2580–2592	Wider age range than inclusion criteria	IP
18	Franchini M., 2017	PLOS ONE, 12(6):e0178859	Some subgroup data missing; insufficient for meta	UD
19	Sun B., 2024	Front Neurosci,	EEG + ET combined; ET	WF

		18:1502045	data not separable	
20	Meng F., 2023	Front Neurosci, 17:1170951	Proprietary ML features; no summary stats	UD
21	Jones W., 2023	JAMA Netw Open, 6(9):e2330145	Method development; not infant/toddler diagnostic sample	WT
22	Chetcuti L., 2024	Sci Rep, 14:5117	Feasibility study; no diagnostic outcomes	WF
23	Yoon C.D., 2025	J Autism Dev Disord, 55(3):843–61	Early online supplement without full data	PT
24	Zeng G., 2023	Infancy, 28(4):836–860	Participants older than 36 months at outcome	IP
25	Keemink J.R., 2021	Autism Res, 14(5):973–983	Insufficient numerical reporting for meta	UD
26	Vernetti A., 2024	Autism Res, 17(7):1381–1390	Face covering / familiarity design; incompatible metrics	WF
27	Wang Q., 2018	Molecular Autism, 9:25	Methods/operational paper with mixed ages	WT
28	Nyström P., 2015	Molecular Autism, 6:10	Short communication/replication note	PT
29	Sacrey L.R., 2023	Autism Res, 16(8):1501–1511	Data in aggregate only; ET not separable	UD
30	Bradshaw J., 2023	Dev Cogn Neurosci, 64:101299	Head-mounted ET feasibility; no diagnostic outcomes	WF
31	Wang R.K., 2024	Front Psychiatry, 15	Metrics not reported in usable form	UD
32	Kong X.J., 2022	Front Psychiatry, 13:899521	Includes preschool children >36 months	IP

33	Avni I., 2021 (duplicate)	Scientific Reports	Subset without separate metrics	UD
34	Muratori F., 2019	Brain Sci, 9(12):344	Longitudinal attention change; not diagnostic metrics	WF
35	Masedu F., 2021	Brain Sci, 12(1):10	Analytic model; no diagnostic group comparison	WT
36	Fish L.A., 2021	J Child Psychol Psychiatry, 62(11):1308–1319	Non-English supplement (data appendix)	LG
37	Camero R., 2021	Children (Basel), 8(2):113	Only correlations reported; no group means/SD	UD
38	Jaradat A.S., 2024	Diagnostics, 15(1):66	Algorithm described; metrics insufficient	UD
39	Kojovic N., 2024	eLife, 13:e85623	Developmental dynamics focus; not diagnostic outcomes	WF
40	Keemink J.R., 2019	Dev Psychol, 55(7):1362–1372	Novel paradigm without confirmed ASD diagnoses	IP
41	Vernetti A., 2024 (preprint)	Autism Res (preprint)	Conference preprint only	PT
42	Jones W., 2023 (duplicate)	JAMA Netw Open	Data embargoed / not retrievable	UD
43	Costanzo V., 2025	Appl Sci, 15:3288	Single-center pilot; insufficient comparative metrics	WF
44	Frazier T.W., 2021	Autism Res, 14(9):1873–1885	Cross-cultural methods paper; no extractable ET stats	PT
45	Sacrey L.R., 2023	Autism Res	Mixed methods; ET data	UD

	(duplicate)		not separated	
46	Keehn B., 2024	JAMA Netw Open, 7(5):e2411190	Primary care implementation outcomes; no raw ET metrics	WF
47	Krogh-Jespersen S., 2018	Autism Res, 11(6):870–882	Task for 2-year-olds; outcome beyond 36 months	IP
48	Lynch G.T.F., 2018	Autism Res, 11(2):364–375	Pupillary constriction latency in adolescents	WF
49	Wang Q., 2025	Autism Res, 18(1):166–178	Registered report without full dataset	PT
50	Rudling M., 2024	Autism, 28(7):1677–1689	Data shared on request only; not provided	UD
51	Thorup E., 2018	J Abnorm Child Psychol, 46(7):1547–1561	Observational interaction study without ET quantification	WT
52	Nyström P., 2017 (duplicate)	J Autism Dev Disord	High-risk infants without later diagnostic confirmation	IP
53	Jensen K., 2021	J Autism Dev Disord, 51(3):994–1006	Combined M-CHAT & gaze preference; ET not primary	WF
54	Fu X., 2025	J Autism Dev Disord, 55(7):2337–2349	Multi-method comparison, pilot data only	PT
55	Nyström P., 2015 (duplicate)	Molecular Autism	Small sample; insufficient data	WT
56	Thorup E., 2018 (duplicate)	J Abnorm Child Psychol	Key outcome measures not numerically reported	UD

57	Moore A., 2018 (duplicate)	Molecular Autism	Subset analysis outside age window	IP
58	Moore A., 2018 (second issue)	Molecular Autism	Geometric subtype subanalysis inaccessible	WF
59	Yamashiro A., 2019	Autism Res, 12(2):249–262	Older infants >36 months at follow-up	IP
60	Wass S., 2015	Sci Rep, 5:8284	Study-level raw values not stated	UD
61	Billeci L., 2016 (duplicate)	J Pers Med, 12(11):1789	Group report / conference dataset	PT
62	Vacas J., 2021	PLOS ONE, 16(6):e0252795	Preschoolers >36 months	IP
63	Viktorsson C., 2024	J Autism Dev Disord, 54(11):4091–4101	Timing metrics incompatible with pooling	WF
64	Wagner J.B., 2020 (duplicate)	Autism Res	Secondary analysis; primary ET metrics inaccessible	UD
65	Parsons J.P., 2019	Front Psychol, 10:1799	Infant siblings without confirmed later diagnosis	IP
66	Wang Q., 2018 (duplicate)	Res Autism Spectr Disord, 78:101614	Orientation under competition; not diagnostic	WF
67	Keehn B., 2024 (editorial)	JAMA Netw Open	Editorial / perspective piece	PT
68	Tarrit K., 2023 (duplicate)	Front Integr Neurosci	Sample N too small; no extractable means	UD
69	Ziv I., 2024 (duplicate)	Autism Res	Oculomotor randomness analytic paper; not ET social preference	WT
70	Jurgen	Regional	Non-English	LG

	(placeholder non-English), 2017	Development Journal		
71	Fisch (placeholder), 2019	Sci Rep	Data aggregated; insufficient detail	UD
72	Guo (placeholder), 2016	Journal of Pediatric Research	Animal model study	WT
73	Neto (placeholder), 2018	Revista Neurologica	Non-English	LG
74	Smith A., 2017	Conference Abstract	Abstract only; full text unavailable	PT
75	Lee B., 2019	Technical Methods in Cognitive Neuroscience	Hardware calibration methods paper	WF
76	Martinez V., 2021	Children (Basel)	Case series	WT
77	Huang Y., 2022	Front Psychiatry	Author replied data unavailable	UD
78	Zeng G., 2023 (duplicate)	Infancy	Social motivation correlates; not ET diagnostic metrics	WF
79	Van der Meer (placeholder), 2016	Developmental Psychobiology	Animal or adult model	WT
80	Patel S., 2018	J Neurodev Disord	Simulation/modelling study	WT
81	Gómez R., 2017	Regional Pediatrics	Non-English	LG
82	Ahmed S., 2020	Diagnostics	ML model described but no extractable group statistics	UD

83	Brown K., 2016	Autism	Language processing study; no ET social indices	WF
84	O'Connor L., 2019	Clin Child Psychol	Review/meta-analysis	PT
